# Golgi-mediated microtubule nucleation is associated with initiation of vertebrate peripheral neuron regeneration

**DOI:** 10.1016/j.isci.2025.113697

**Published:** 2025-10-04

**Authors:** Alice E. Mortimer, Adam J. Reid, Raman M. Das

**Affiliations:** 1 Blond McIndoe Laboratories, Division of Cell Matrix Biology and Regenerative Medicine, School of Biological Sciences, Faculty of Biology, Medicine and Health, Manchester Academic Health Science Centre, The University of Manchester, Manchester M13 9PT, UK; 2 Department of Plastic Surgery and Burns, Manchester University NHS Foundation Trust, Manchester Academic Health Science Centre, Wythenshawe Hospital, Manchester M23 9LT, UK; 3 Division of Molecular and Cellular Function, School of Biological Sciences, Faculty of Biology Medicine and Health, Manchester Academic Health Science Centre, The University of Manchester, Manchester M13 9PT, UK

**Keywords:** molecular biology, neuroscience, cell biology

## Abstract

Peripheral neurons have the potential to regenerate following injury, yet a poor understanding of the cell intrinsic drivers of this process have prevented clinical exploitation. Using *in vitro* and *in vivo* models, we define a conserved mechanistic basis for initiation of adult human and rat peripheral neuron regeneration that highlights the Golgi as a major driver of peripheral neuron regeneration. Acute injury first induces somatic Golgi fragmentation followed by rapid re-compaction as a pre-requisite to regeneration. Initiation of axon regeneration is then triggered through transient stepwise recruitment of the microtubule nucleation factors AKAP9 and γ-tubulin, resulting in Golgi-mediated microtubule nucleation. Consequently, disruption of Golgi compaction or AKAP9 and γ-tubulin recruitment compromises induction of microtubule nucleation and initiation of regeneration. This work redefines our understanding of the conserved cell-intrinsic mechanisms initiating peripheral neuron regeneration and identifies Golgi-mediated microtubule nucleation as a key therapeutic target in an area of clinical unmet need.

## Highlights

• Peripheral neuron injury induces Golgi fragmentation followed by re-compaction
• AKAP9 and γ-tubulin recruitment leads to Golgi-mediated microtubule nucleation
• Inhibition of Golgi compaction or recruitment of AKAP9 inhibits axon regeneration
• These mechanisms are conserved between rat and human species

## Graphical abstract

**Figure:**
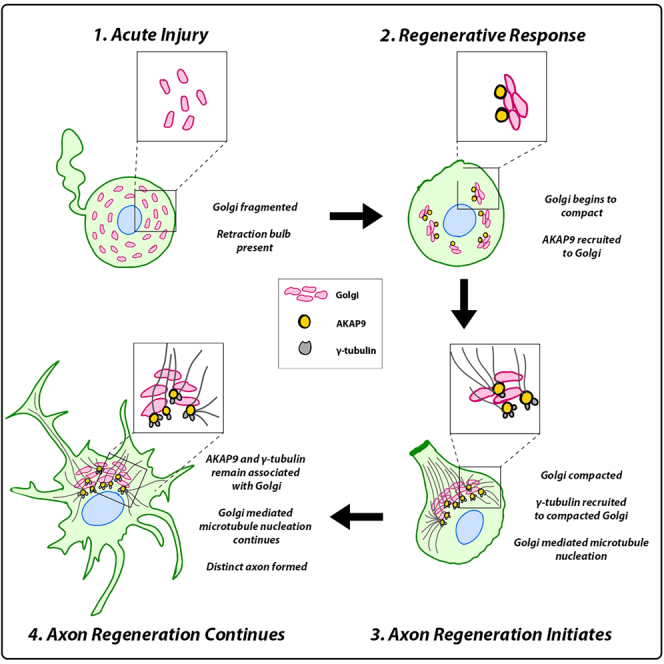

## Introduction

Clinical reliance upon innate regenerative capacity of injured peripheral neurons is never sufficient for meaningful restoration of function in major nerves. Regeneration following peripheral nerve injury (PNI) requires robust initiation and extension of axons upwards of one meter to reach the target tissue.^1^ Current clinical interventions are restricted to surgical repair at the site of injury to provide a permissive environment for regeneration; however, not all peripheral neurons initiate regeneration equally in response to injury.^1^^,^^2^^,^^3^ Indeed, the fundamental cellular mechanisms driving peripheral neuron regeneration remain unknown, impeding the development of new strategies to accelerate this process.

Axon extension is dependent on rearrangement of the neuronal cytoskeleton, of which microtubules are essential constituent playing vital roles in cellular structure, determining cell polarity, and allowing for bidirectional cargo transport.^4^ Following PNI, nascent microtubule nucleation presumably drives axon regeneration, yet mature neurons lack a functional centrosome, which acts as the main microtubule organizsing center (MTOC) in most dividing cells including during axon initiation in differentiating mammalian neurons.^5^ Therefore, an alternative cellular compartment must be responsible for microtubule nucleation in regenerating adult peripheral neurons, but no other microtubule nucleating platforms have yet been identified. Several distinct subcellular compartments have been demonstrated to possess MTOC capacity, including the plasma membrane, the nuclear membrane, and the Golgi apparatus.^6^^,^^7^^,^^8^^,^^9^ In addition, the Augmin/HAUS complex has been demonstrated to facilitate polarized microtubule nucleation from pre-existing microtubules in maturing neurons^10^^,^^11^ and plays a role in stabilizing dendrites in *Drosophila* neurons.^12^^,^^13^ Golgi-mediated microtubule nucleation often coordinates with centrosomal microtubule nucleation in several cellular contexts.^14^^,^^15^^,^^16^^,^^17^ In developing cortical neurons, centrosomal microtubules drive neuron migration while Golgi-derived microtubules support axonal growth.^18^^,^^19^ Localized microtubule nucleation from Golgi outposts has also been implicated in shaping cells with unusual morphologies, including neurons and oligodendrocytes,^17^ and the Golgi has been implicated as a microtubule nucleating platform in *Drosophila* larval neurons^16^ and regenerating *Drosophila* quiescent neural stem cells.^20^ In addition, the somatic Golgi has a well-described interplay with microtubules as a means of fulfilling vital roles in polarized cell transport and migration.^21^^,^^22^ Despite this, there is no precedent for Golgi mediated microtubule nucleation acting as the major driver of a complex clinically relevant morphological transformation such as initiation of vertebrate axon regeneration.

Here, we report a conserved neuronal mechanism that promotes initiation of axon regeneration in response to PNI across rat and human species, *in vitro* and *in vivo.* Using adult dorsal root ganglia (DRG) sensory neuron injury models which addresses a major unmet clinical challenge to restore sensory function, we observe injury induced somatic Golgi fragmentation followed by rapid compaction. This facilitates transient stepwise recruitment of the key microtubule nucleating factors AKAP9 and γ-tubulin during a distinct period that corresponds with initiation of axon regeneration. Disruption of Golgi compaction prevents recruitment of AKAP9 and γ-tubulin, leading to a reduction in microtubule nucleation and failure of axon regeneration. Furthermore, disruption of AKAP9 recruitment to the Golgi results in reduced γ-tubulin recruitment and ultimately compromises the innate ability to regenerate axons following neuronal injury. These results together demonstrate that compaction of the Golgi and AKAP9-mediated γ-tubulin recruitment to the compacted Golgi drives microtubule nucleation following PNI to trigger initiation of axon regeneration.

## Results

### Peripheral neuron injury induces Golgi fragmentation and subsequent compaction leading to emergence of microtubules from the Golgi

**Figure 1:**
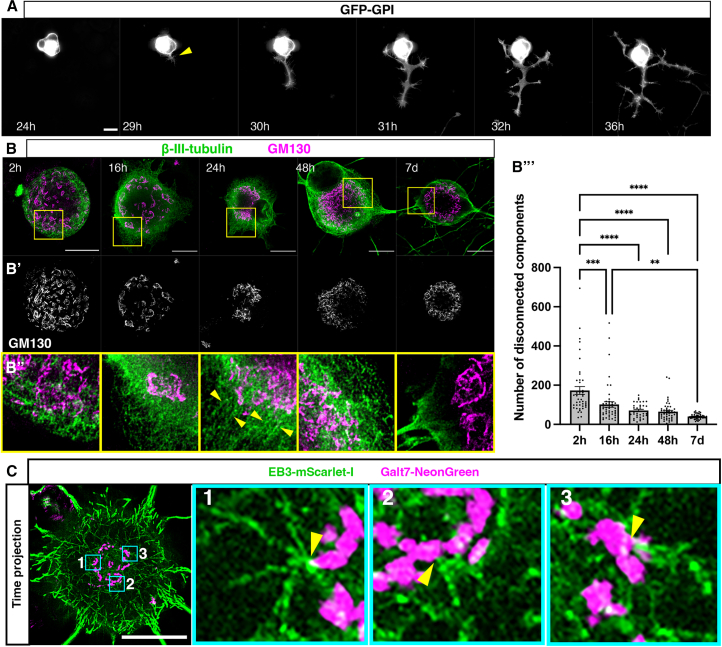
Golgi compaction is associated with emergence of microtubules from the Golgi and initiation of axon regeneration (A) Time-lapse sequence of dissociated DRG neuron expressing GFP-GPI initiating axon regeneration at 29 h post-injury (yellow arrowhead). (B) Immunostaining to detect β-III-tubulin and GM130 from 2 h up to 7 days post-injury, imaged using super-resolution STED microscopy. (B’) GM130 shown in grayscale, highlighting the changes in Golgi morphology from 2 h to 7 days post-injury. Yellow boxes in (B) outline zoomed regions in (B’’), where linear tracks of microtubules appear to emerge from the Golgi at 24 h (yellow arrowheads). (B’’’) Quantification of mean number of disconnected components in the GM130 channel: 173 ± 20.26 at 2 h (*n* = 44 cells), 101.5 ± 13.94 at 16 h (*n* = 50 cells), 70.94 ± 6.12 at 24 h (*n* = 36 cells), 65.02 ± 6.72 at 48 h (*n* = 49 cells) and 40.19 ± 1.97 at 7 days (*n* = 43 cells). *N* = 3 animals throughout. *p* = 0.0003 2 vs. 16 h; *p* < 0.0001 2 h vs. 24 h, 48 h, and 7 days; *p* = 0.003 16 h vs. 7 days. (C) Time projections of dissociated DRG neuron at 36 h post-injury transfected with Galt7-NeonGreen and EB3-mScarlet-I. Cyan boxes outline zoomed regions 1, 2, and 3. Yellow arrowheads indicate sites of nucleation. Scale bars: 20 μm. All graphs displayed as mean ± SEM,∗*p* < 0.05, ∗∗*p* < 0.01, ∗∗∗*p* < 0.001, ∗∗∗∗*p* < 0.0001; ordinary one-way ANOVA and Tukey’s post hoc test used for statistical analyses.

To investigate the cellular dynamics of axon regeneration following PNI, we induced acute axotomy *in vitro* by dissecting adult rat DRGs from the animal, followed by enzymatic and mechanical dissociation to obtain single neurons. Cells were then cultured and transfected with a plasmid expressing GFP-GPI to label cell membranes and subjected to live imaging using high-resolution widefield timelapse microscopy from 24 h post-injury. This revealed onset of axon regeneration at 24–30 h post-injury (21/31 cells, 8 animals). During time-lapse imaging up to 40 h post-injury, 14/31 cells established a dominant axonal extension, and 7/31 cells established the typical pseudo-unipolar morphology of DRG neurons (Figure 1A, Video S1). To confirm this and to investigate Golgi dynamics during peripheral neuron regeneration, dissociated cells were fixed at 2, 16, 24, 48 h, and 7 days post-injury and labeled for β-III-tubulin to label neuron specific microtubules and the cis Golgi marker GM130 (Figures 1B and 1B′). At 2 h post-injury, the Golgi was fragmented and dispersed within the soma. Over the course of regeneration, the Golgi then compacted in the soma, most notably in the first 24 h. Golgi compaction then stabilized between 24 h and 7 days. These observations were confirmed by quantification of the number of discrete components present in the GM130 channel in 3D which were not in contact with other GM130 positive components, hereafter referred to as “disconnected” components (Figure 1B’’’; 3 animals, 44 cells at 2 h, 50 cells at 16 h, 36 cells at 24 h, 49 cells at 48 h, and 43 cells at 7 days). To characterize how these conformational changes in the Golgi related to the stages of regeneration we determined the number of cells at 2, 16, 24, 48 h, and 7 days post-injury exhibiting various hallmarks of regeneration (Figure S1; 3 animals). At 2 h most cells either possessed clear retraction bulbs (38/76 cells), an indication of acute injury, or had no extensions (37/76) while a single cell displayed an asymmetric reduction in microtubule labeling that we term the “microtubule penannular” (Figure S1A). At 16 h there was a reduction in the number of cells with retraction bulbs (24/90) or with no extensions (27/90), while the number of cells with a microtubule penannular increased (36/90). A small number of these (3/90) now also possessed multiple extensions. At 24 h there was a further reduction in cells with retraction bulbs (16/102) or no extensions (17/102), and a corresponding increase in cells with a microtubule penannular (60/102) or multiple extensions (5/102). Furthermore, a small number of these cells now displayed a dominant axon (4/102). This trend continued at 48 h, with no cells displaying a retraction bulb (0/80), fewer cells with no extensions (2/80) or a microtubule penannular (6/80). Conversely, there was an increase in cells with multiple extensions (43/80) or a dominant axon (29/80). By 7 days most cells either displayed multiple extensions (32/62) or a dominant axon (30/62) (Figure S1B). We then correlated these morphological hallmarks of regeneration with the state of Golgi compaction in these cells. This revealed that cells with clear retraction bulbs exhibited the greatest number of disconnected Golgi components, closely followed by cells with no extensions and cells at the microtubule penannular stage. In contrast, cells with multiple extensions or with a dominant axon displayed a sharp decrease in the number of disconnected Golgi components, demonstrating a clear correlation between Golgi compaction and initiation of axon regeneration (quantified in Figure S1C).

We further observed using super-resolution stimulated emission depletion (STED) microscopy (Figure 1B’’) that microtubules exhibited a disorganized appearance at 2- and 16-h post-injury. Conversely, at 24 h, when axon regeneration initiates, linear tracks of microtubules were visualized that characteristically appeared to emerge from the Golgi, suggesting that the compacted Golgi may be acting as a platform for microtubule nucleation at this time point. It appeared, however, that this was transient, as somatic microtubules appeared to return to their disorganized state at 48 h and 7 days post-injury. To determine the origin of nascent microtubules in regenerating peripheral neurons, we monitored microtubule nucleation patterns by labeling microtubule plus-ends with EB3-mScarlet-I and the Golgi with Galt7-NeonGreen in dissociated DRG neurons *in vitro*. Live imaging at 36 h post-injury, when expression of transfected constructs was established, revealed microtubule comets emerging from the Golgi in a radial pattern, with no apparent polarization (Figure 1C, Video S2; 13 cells, 4 animals). Furthermore, time projections facilitated visualization of microtubule tracks (Figure 1C, far right panel), confirming their origin from the Golgi. Taken together, these results indicate that Golgi compaction corresponds with Golgi-mediated microtubule nucleation during initiation of peripheral neuron regeneration *in vitro*.

### Microtubule nucleation factors AKAP9 and γ-tubulin associate with the Golgi during initiation of axon regeneration

**Figure 2:**
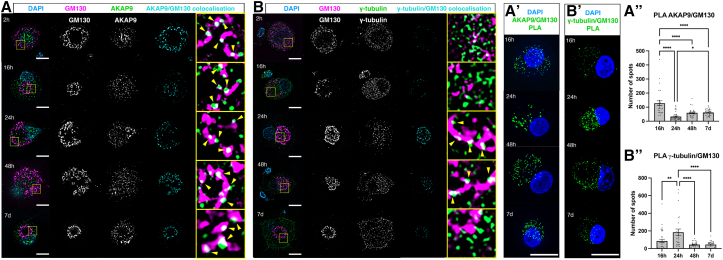
Sequential recruitment of AKAP9 and γ-tubulin to the Golgi (A) Immunolabeling of dissociated DRG neurons 2 h to 7 days post-injury for GM130 and AKAP9. Yellow arrowheads: AKAP9 puncta decorating Golgi. Yellow boxes outline zoomed in regions. Cyan channel displays regions of colocalization between AKAP9 and GM130. (A’) PLA to determine interactions between GM130 and AKAP9 at 16 h to 7 days. (A’’) Quantification of PLA puncta: 127.3 ± 17.52 at 16 h (*n* = 27 cells); 32.68 ± 4.29 at 24 h (*n* = 40 cells); 59.55 ± 5.62 at 48 h (*n* = 29 cells) and 61.76 ± 3.48 at 7 days (*n* = 37 cells). *N* = 2 animals throughout. *p* < 0.0001 16 vs. 24 h, 48 h and 7 days; *p* = 0.0381 24 h vs. 7 days. (B) Immunolabeling of dissociated DRG neurons 2 h to 7 days post-injury for GM130 and γ-tubulin. Yellow arrowheads: γ-tubulin puncta decorating Golgi. Yellow boxes outline zoomed in regions. Cyan channel displays regions of colocalization between γ-tubulin and GM130. (B’) PLA to determine interactions between GM130 and γ-tubulin at 16 h to 7. (B’’) Quantification of PLA puncta: 85.81 ± 20.64 at 16 h (*n* = 32 cells), 184.2 ± 37.75 at 24 h (*n* = 25 cells), 44.48 ± 5.97 at 48 h (*n* = 27 cells) and 44.96 ± 7.07 at 7 days (*n* = 28 cells). *N* = 2 animals throughout. *p* = 0.0069 16 h vs. 24 h; *p* < 0.0001 24 h vs. 48 h and 7 days. Scale bars: 20 μm. All graphs display mean ± SEM. ∗*p* < 0.05, ∗∗*p* < 0.01, ∗∗∗∗*p* < 0.0001; ordinary one-way ANOVA and Tukey’s post hoc test used for statistical analyses.

Golgi-mediated microtubule nucleation is facilitated by recruitment of AKAP9 and γ-tubulin to the cis-Golgi membrane in several experimental systems, including cultured cells and Drosophila neurons.^16^^,^^23^^,^^24^^,^^25^^,^^26^ To investigate if compaction of the fragmented Golgi acts as a key temporal determinant of AKAP9 and γ-tubulin recruitment to drive adult vertebrate peripheral neuron regeneration, we labeled dissociated rat DRG neurons for the cis-Golgi protein GM130, AKAP9, and γ-tubulin. This revealed AKAP9 puncta associated with the Golgi at all time-points from 2 h to 7 days post-injury, suggesting recruitment of AKAP9 to the Golgi at all stages (Figure 2A; 115 cells at 2 h, 128 cells at 16 h, 88 cells at 24 h; 46 cells at 48 h, 51 cells at 7 days; 3 animals). In contrast, labeling for γ-tubulin and GM130 revealed that although γ-tubulin was expressed at all time points, there was no apparent association with the Golgi at 2 h, 16 h and 7 days (Figure 2B; 62 cells at 2 h, 117 cells at 16 h, 151 cells at 7 days; 3 animals). Strikingly, we observed clear association at 24 and 48 h, which, respectively, correspond with Golgi compaction (Figure 2A; 85 cells at 24 h, 51 cells at 48 h; 3 animals) and emergence of microtubules from the Golgi (Figure 1C), suggesting that recruitment of γ-tubulin at these specific time points may facilitate Golgi-mediated microtubule nucleation.

To confirm interactions between GM130, AKAP9, and γ-tubulin from 16 h to 7 days post-injury we performed proximity ligation assays (PLA), which is a method for visualizing endogenous protein interactions using primary antibodies which generates fluorescent puncta when two proteins of interest are within close proximity to each other.^27^ This confirmed recruitment of AKAP9 to the Golgi at all stages (Figure 2A’ and 2A’’; 27 cells at 16 h, 40 cells at 24 h, 29 cells at 48 h, 37 cells at 7 days; 2 animals). Notably, we observed minimal recruitment of γ-tubulin to the Golgi at 16 h, robust recruitment at 24 h post-injury followed by a sharp decline in recruitment at 48 h which persisted until 7 days post-injury (Figure 2B’ and 2B’’; 32 cells at 16 h, 25 cells at 24 h, 27 cells at 48 h, 28 cells at 7 days; 2 animals). The specificity of these experiments was confirmed by performing PLA on cells fixed at 24 h post-injury that had not been labeled for GM130 and AKAP9 or γ-tubulin, which resulted in no detectable PLA puncta (Figure S2; 24 cells; 2 animals). These observations demonstrate that Golgi compaction following peripheral neuron injury is characterized by recruitment of AKAP9 to the compacting Golgi, which is followed by recruitment of the key microtubule nucleation factor γ-tubulin during the period corresponding with initiation of axon regeneration.

### The mechanisms characterizing initiation of axon regeneration *in vitro* are conserved *in vivo* and in human peripheral neurons

**Figure 3:**
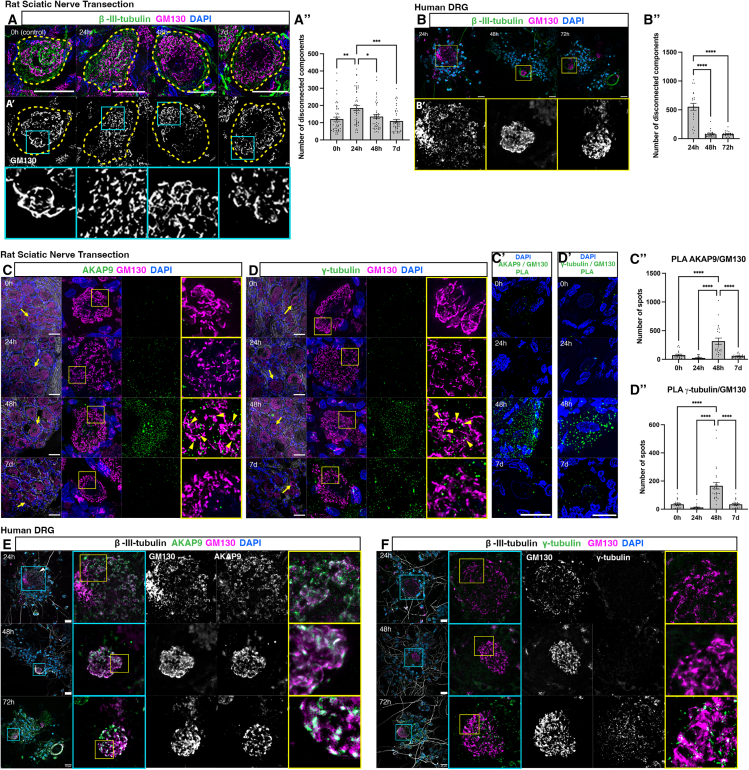
The mechanisms promoting initiation of axon regeneration *in vitro* are conserved *in vivo* and in human peripheral neurons (A) Immunolabeling of tissue sections from L4/5 rat DRGs following sciatic nerve transection and subsequent recovery for 0 h, 24 h, 48 h, and 7 days for β-III-tubulin and GM130. Yellow dashed lines outline individual cells. (A’) GM130 shown in grayscale demonstrating fragmentation at 24 h post-injury followed by compaction to baseline (control) from 48 h to 7 days. Cyan boxes outline zoomed regions in the bottom panel. (A’’) Quantification of mean number of disconnected components in GM130 channel: 121 ± 11.95 at 0 h (*n* = 48 cells), 184.6 ± 3.97 at 24 h (*n* = 50 cells), 135.7 ± 11.94 at 48 h (*n* = 44 cells) and 109.7 ± 9.95 at 7 days (*n* = 46 cells). *N* = 3 animals. *p* = 0.0012 0 h vs. 24 h, *p* = 0.0251 24 h vs. 48 h, *p* = 0.0001 24 h vs. 7 days. (B) Dissociated human DRG labeled for β-III-tubulin and GM130. Yellow boxes outline zoomed regions in (B’) GM130 shown in grayscale, highlighting the changes in Golgi morphology from 24 h to 72 h post-injury. (B’’) Quantification of mean number of disconnected components in GM130 channel: 553.1 ± 60.07 at 24 h (*n* = 21 cells), 75 ± 8.93 at 48 h (*n* = 34 cells) and 80.33 ± 9.65 at 72 h (*n* = 21 cells). *N* = 3 human donors. p=<0.0001 24 h vs. 48 h, p=<0.0001 24 h vs. 72 h. (C) Immunolabeling of L4/5 rat DRGs tissue sections following sciatic nerve transection and recovery for 0/24/48 h and 7 days for GM130 and AKAP9. Yellow arrowheads: AKAP9 puncta decorating Golgi. Zoomed in regions identified by yellow arrows/boxes. (C’) PLA to determine interactions between GM130 and AKAP9 at 0 h to 7 days post-injury. (C’’) Quantification of PLA puncta: 74.9 ± 16.13 at 0 h (*n* = 20 cells), 22.67 ± 6.23 at 24 h (*n* = 21 cells), 316.8 ± 53.37 at 48 h (*n* = 23 cells) and 53.91 ± 6.13 at 7 days (*n* = 23 cells). *N* = 2 animals. *p* < 0.0001 48 h vs. 0 h, 24 h and 7 days. (D) Immunolabelling of tissue sections for GM130 and γ-tubulin. Yellow arrowheads: γ-tubulin puncta decorating Golgi. (D’) PLA to determine interactions between GM130 and γ-tubulin at 0 h to 7 days post-injury. (D’’) Quantification of PLA puncta: 34.32 ± 6.16 at 0 h (*n* = 22 cells), 1.6 ± 3.51 at 24 h (*n* = 20 cells), 166.3 ± 23.09 at 48 h (*n* = 28 cells) and 34.32 ± 6.16 at 7 days (*n* = 22 cells). *N* = 2 animals. *p* < 0.0001 48 h vs. 0 h, 24 h and 7 days. (E) Dissociated human DRG fixed at 24/48/72 h post-injury, labeled for GM130 and AKAP9. Zoomed in regions enclosed in blue/yellow boxes. (F) Dissociated primary human DRG fixed at 24/48/72 h post-injury, labeled for GM130 and γ-tubulin. Zoomed in regions enclosed in blue/yellow boxes. Scale bars: 20 μm. All graphs display mean ± SEM. ∗*p* < 0.05, ∗∗*p* < 0.01, ∗∗∗∗*p* < 0.0001; ordinary one-way ANOVA and Tukey’s post hoc test used for statistical analyses.

To confirm the physiological relevance of our findings, we performed *in vivo* unilateral sciatic nerve transections at the level of the proximal femur to effect axotomy approximately 4 mm from the L4/L5 DRG, which are the major contributors to the sciatic nerve in adult rats. We further hypothesized that performing nerve injury distal to the DRG cell bodies would result in a slower regenerative response and therefore present a greater opportunity to study the temporal dynamics of the mechanisms characterizing initiation of axon regeneration. Animals underwent terminal anesthesia and immediate isolation and fixation of L4/L5 DRG following post-injury survival periods of 0, 24, 48 h, and 7 days. Fixed DRG were sectioned and labeled with β-III-tubulin and GM130 (Figures 3A and 3A’; 3 animals, 48 cells at 0 h, 50 cells at 24 h, 44 cells at 48 h, 46 cells at 7 days). This revealed that the Golgi *in vivo* exhibited similar responses to injury as dissociated neurons *in vitro*. The control condition where DRG were immediately extracted from the animal and fixed (0 h) provided an indication of Golgi morphology in homeostatic DRG neurons and facilitated comparison with DRG neurons undergoing regeneration following injury. This revealed that the Golgi was compacted within the soma in uninjured neurons at 0 h. In contrast, by 24 h post-injury a dispersal pattern was evident similar to that observed at 2 h *in vitro*. By 48 h, the Golgi had re-compacted to levels similar to the 0-h control and remained as such until 7 days. These observations were confirmed by disconnected component analysis (Figure 3A’’), with 64% of cells at 24 h post-injury displaying higher levels of Golgi fragmentation compared to the mean levels of fragmentation at 0 h (32/50 cells). This was followed by a reduction to 50% of cells at 48 h post-injury (22/44 cells) and 28% of cells at 7 days post-injury (13/46 cells). Therefore, DRG neuron Golgi *in vivo* exhibit a similar response to injury as dissociated neurons *in vitro*, but the *in vivo* response proceeds at a slower pace.

We then explored the clinical relevance of these findings by fixing and labeling human donor-derived DRG neurons *in vitro* with β-III-tubulin and GM130 at 24, 48, and 72 h post-injury (Figure 3B and 3B’; 3 human donors, 21 cells at 24 h, 34 cells at 48 h, 21 cells at 72 h). As human derived DRG neurons are a limited resource, we based these time points on our previous observation in rats *in vivo*. This revealed similar Golgi dynamics to those observed in rat DRGs *in vitro* and *in vivo*. The Golgi was fragmented and dispersed within the soma at 24 h post-injury followed by compaction at 48 h post-injury and maintenance of Golgi compaction at 72 h post-injury (quantified in Figure 3B’’). These findings indicate that the pattern of injury-induced Golgi fragmentation followed by Golgi compaction is conserved between rats and humans.

In the *in vivo* context of rat sciatic nerve transections, we further observed clear AKAP9 and γ-tubulin association with the Golgi at 48 h post-injury, coinciding with Golgi compaction (Figure 3C), but not at 0 h, 24 h, or 7 days post-injury (Figures 3C and 3D; 48 cells at 0 h, 50 cells at 24 h, 44 cells at 48 h, 46 cells at 7 days post-injury; 3 animals). These associations were confirmed by PLA, which revealed robust recruitment of both AKAP9 (Figures 3C’ and 3C’’; 20 cells at 0 h, 21 cells at 24 h, 23 cells at 48 h, 23 cells at 7 days post-injury; 2 animals) and γ-tubulin (Figures 3D’ and 3D’’; 22 cells at 0 h, 20 cells at 24 h, 28 cells at 48 h, 22 cells at 7 days post-injury; 2 animals) to the Golgi at 48 h post-injury, but not at 0 h, 24 h or 7 days post-injury.

To determine if similar dynamic recruitment of AKAP9 and γ-tubulin takes place in the context of human peripheral neuron regeneration, we fixed and labeled human donor-derived DRG neurons *in vitro* with GM130, AKAP9, and γ-tubulin at 24, 48, and 72 h post-injury. Similar to our findings *in vitro* in rat, we observed clear associations between the Golgi and AKAP9 at all time points (Figure 3E; 46 cells at 24 h, 44 cells at 48 h, 39 cells at 72 h; 3 donors). At 24 and 48 h post-injury, no associations between the Golgi and γ-tubulin were evident (Figure 3F; 47 cells at 24 h, 60 cells at 48 h; 3 donors). However, at 72 h post-injury, we observed a clear association between the Golgi and γ-tubulin puncta (Figure 3F; 48 cells; 3 donors). Taken together, these observations strongly demonstrate that the pattern of transient, stepwise recruitment of AKAP9 and γ-tubulin is conserved between rat and human species but proceeds at a slower pace in the context of human PNI.

### A compacted Golgi is required to trigger microtubule nucleation and promote axon regeneration

**Figure 4:**
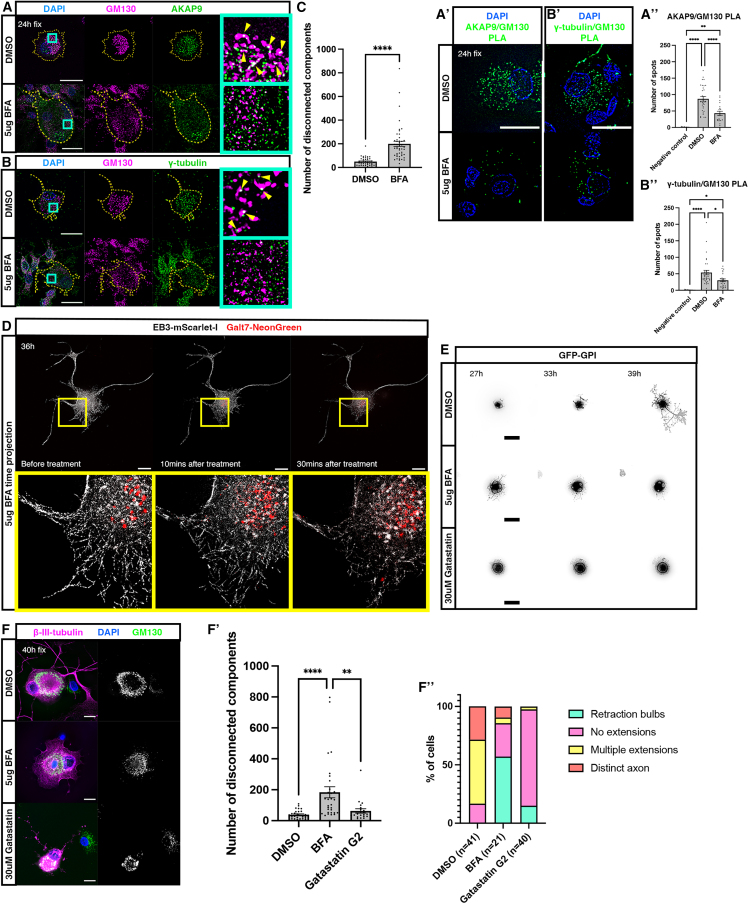
Golgi compaction is required for initiation of axon regeneration (A) Immunostaining to detect GM130 and AKAP9 in dissociated DRG neurons cultured in medium containing DMSO (top panels) or BFA (bottom panels). Yellow arrowheads: AKAP9 puncta decorating Golgi. Blue boxes indicate zoomed in regions shown in right-hand panel. (B) Immunostaining to detect GM130 and γ-tubulin in dissociated DRG neurons cultured in medium containing DMSO (top panels) or BFA (bottom panels). Yellow arrowheads: γ-tubulin puncta decorating Golgi. Blue boxes indicate zoomed in regions shown in right-hand panel. (A’) PLA to determine interactions between GM130 and AKAP9 in dissociated DRG neurons treated with DMSO or BFA. (A’’) Quantification of PLA puncta: 87.06 ± 7.09 DMSO (*n* = 32 cells) and 43.25 ± 6.1 BFA treated cells (*n* = 20 cells). *N* = 2 animals. (B’) PLA to determine interactions between GM130 and γ-tubulin in dissociated DRG neurons treated with DMSO or BFA. B″) Quantification of PLA puncta: 53.56 ± 6.87 DMSO (*n* = 34 cells) and 30.17 ± 4.29 BFA treated cells (*n* = 24 cells). *N* = 2 animals. (C) Quantification of mean number of disconnected components in GM130 channel: 49.56 ± 5.13 in DMSO treated cells (*n* = 30 cells) and 199.3 ± 21.92 in BFA treated cells (*n* = 30 cells). *N* = 2 animals. p=<0.0001. (D) Time projections of 1-min timelapse sequence of cell expressing EB3-mScarlet-I and Galt7-NeonGreen following addition of BFA. Yellow boxes indicate zoomed in regions shown in bottom panels. (E) Timelapse sequences of cells expressing GFP-GPI imaged in medium containing DMSO (top panels), BFA (middle panels) and gatastatin G2 (bottom panels). (F) Immunolabeling of cells cultured in media containing DMSO (top panel), BFA (middle panel) or gatastatin G2 (bottom panel) and fixed at 40 h to detect β-III-tubulin and GM130. (F’) Quantification of mean number of disconnected components in GM130 channel: 38.87 ± 5.1 in DMSO treated cells (*n* = 30 cells), 184 ± 35.9 in BFA treated cells (*n* = 30 cells) and 63.23 ± 4.1 in gatastatin G2 treated cells (*n* = 22 cells). *N* = 2 animals. *p* < 0.0001 DMSO vs. BFA treated cells; *p* = 0.0027 gatastatin G2 treated cells vs. BFA treated cells. (F’’) Quantification of the percentage of cells at observed stages of regeneration for each treatment. *N* = 2 animals. Scale bars: 20 μm. All graphs displayed as mean ± SEM. ∗*p* < 0.05, ∗∗*p* < 0.01, ∗∗∗*p* < 0.001, ∗∗∗∗*p* < 0.0001; ordinary one-way ANOVA and Tukey’s post hoc test and unpaired *t* test used for statistical analyses.

To substantiate the role of the Golgi as an MTOC during peripheral neuron regeneration, we applied Brefeldin A (BFA) which inhibits protein transport from endoplasmic reticulum (ER) to Golgi and so disassembles the Golgi.^28^ In doing so, we assessed whether an intact, compacted Golgi is required for recruitment of AKAP9 and γ-tubulin. BFA was applied to dissociated rat DRG cells at 23 h post-injury, when the Golgi is compacted (Figure 1B’), followed by fixation 1 h later. Labeling for GM130 and AKAP9 or γ-tubulin revealed that application of BFA induced disruption of the Golgi compared to cells grown in medium containing DMSO (Figures 4A and 4B, quantified in Figure 4C; 30 cells BFA treated, 30 cells DMSO treated; 2 animals). Furthermore, induced disruption of the Golgi also resulted in loss of AKAP9 and γ-tubulin association with GM130 (Figures 4A and 4B, far right panels; 37 cells γ-tubulin labeled, 39 cells AKAP9 labeled; 2 animals). These observations were confirmed by PLA for interactions between GM130 and AKAP9 or γ-tubulin (Figures 4A’ and 4B′, quantified in Figures 4A’’ and 4B’’; AKAP9/GM130: 32 DMSO cells, 20 BFA treated cells; 2 animals. γ-tubulin/GM130: 34 DMSO cells, 24 BFA treated cells; 2 animals), indicating that an intact, compacted Golgi is required for recruitment of these key microtubule nucleating factors.

BFA was then applied to cells expressing EB3-mScarlet-I to label microtubule plus-ends and Galt7-NeonGreen to label the Golgi at 36 h post-injury followed by timelapse imaging. This revealed rapid disruption of the Golgi within 10 min and an associated decrease in microtubule polymerization events, evidenced by a gradual loss of EB3 comets at 10 min and 30 min following application of BFA (Figure 4D, Video S3; 3 animals, 8 cells). Longer-term timelapse imaging of cells expressing GFP-GPI immediately after addition of BFA over 15 h revealed that disruption of the Golgi resulted in errors in axon regeneration in the majority of cells imaged (15/16 cells, 2 animals) (Figure 4E, Video S4). Of these, 10/16 were unable to initiate axon regeneration (Video S4) and 5/16 possessed axons at the point of BFA application, all of which subsequently retracted toward the cell body. To confirm these findings in cells that were not subjected to our live-imaging regime, we fixed BFA treated cells at 40 h post-injury and labeled for GM130 and β-III-tubulin (Figure 4F). This again revealed disruption of the Golgi compared to DMSO treated cells, indicating that the effects of BFA application persist at 16 h post-application (quantified in Figure 4F’; BFA treated: 30 cells; DMSO treated: 30 cells; 2 animals). Furthermore, most BFA treated cells displayed errors in axon regeneration (18/21 cells, 2 animals). Of these, 6/21 exhibited no regeneration, 12/21 had retraction bulbs present in neurites, 1/21 had multiple extensions and 2/21 had an obvious axon (Figure 4F’’).

To confirm that these effects on axon regeneration were caused by reduced microtubule nucleation, and to discount the effect of impaired Golgi-mediated trafficking, we applied the γ-tubulin functional inhibitor Gatastatin G2.^29^ Crucially, in contrast to BFA treated cells, the Golgi remained compacted in these cells (Figure 4F, quantified in Figure 4F’) suggesting that ER to Golgi trafficking was unaffected and that the concentration of Gatastatin used was not affecting α- or β-tubulin and therefore microtubules themselves, which are required for maintenance of Golgi structure.^30^ Of these Gatastatin treated cells, 33/40 cells exhibited no regeneration, 6/40 had retraction bulbs present, 1/40 had multiple extensions, and 0/40 had an obvious axon (Figure 4F’’). This contrasted with cells grown in medium containing DMSO, in which 7/42 exhibited no regeneration, 0/42 had retraction bulbs, 23/42 multiple extensions and 12/42 an obvious axon (Figure 4F’’). Longer-term timelapse imaging revealed that application of gatastatin over a 15-h period resulted in disrupted axon regeneration, where all treated cells were unable to initiate axonal outgrowth (11/11 cells, 2 animals) (Figure 4E, Video S5). This contrasted with cells imaged in medium containing DMSO, the majority of which were able to undergo axon regeneration (15/23 cells, 2 animals) (Figure 4E, Video S6). These results strongly suggest that an intact, compacted Golgi is a prerequisite for recruitment of AKAP9 and γ-tubulin, which facilitates Golgi-mediated microtubule nucleation that promotes initiation of axon regeneration following injury.

### Golgi associated AKAP9 is required for recruitment of γ-tubulin to promote initiation of axon regeneration

**Figure 5:**
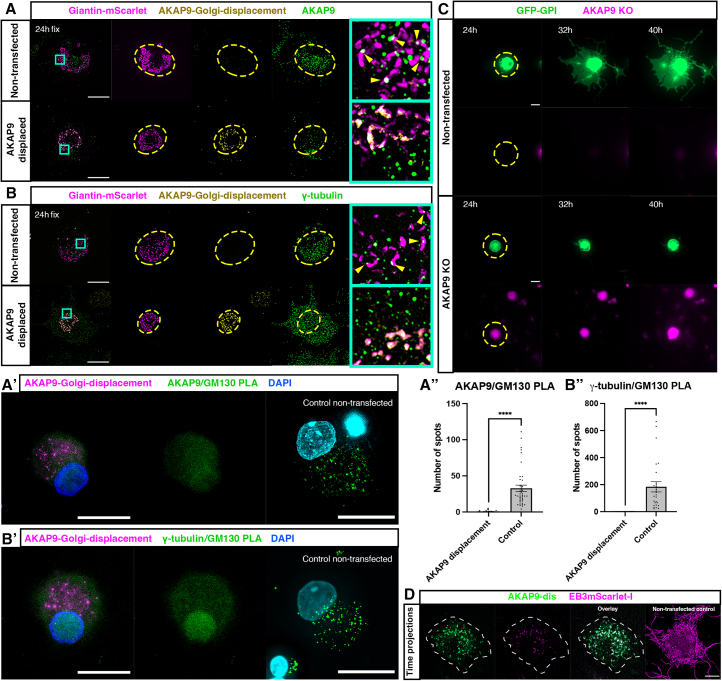
AKAP9 is required for recruitment of γ-tubulin to the Golgi and subsequent axon regeneration (A) Cells fixed at 24 h expressing cis/medial Golgi marker Giantin-mScarlet and AKAP9-dis and immunostained for endogenous AKAP9. Top panel shows control cells not expressing AKAP9-dis and bottom panel cells expressing AKAP9-dis. Yellow dashes outline the area in the cell occupied by the Golgi and cyan boxes outline zoomed in regions. Yellow arrowheads: AKAP9 puncta decorating Golgi. (A’) PLA to determine interactions between GM130 and AKAP9 at 24 h, with non-transfected control cells in the rightmost panel. (B) Cells fixed at 24 h expressing Giantin-mScarlet, AKAP9-dis, and immunostained for endogenous γ-tubulin. Top panel shows control cells not expressing AKAP9-dis and bottom panel cells expressing AKAP9-dis. Yellow dashes outline the area in the cell occupied by the Golgi and cyan boxes outline zoomed regions. Yellow arrowheads: γ-tubulin puncta decorating Golgi. (B’) PLA to determine interactions between GM130 and γ-tubulin at 24 h, with control cells in the rightmost panel. (A’’) Quantification of PLA puncta: 0.58 ± 0.205 in cells transfected with AKAP9-Golgi displacement (*n* = 56 cells) and 32.85 ± 4.4 control non-transfected cells (*n* = 88 cells). *N* = 2 animals. *p* < 0.0001 transfected vs. non-transfected controls. (B’’) Quantification of PLA puncta: 0.143 ± 0.078 in cells transfected with AKAP9-Golgi displacement (*n* = 27 cells) and 184.2 ± 37.75 in control non-transfected cells (*n* = 85 cells). *N* = 2 animals. *p* < 0.0001 transfected vs. non-transfected controls. (C) Timelapse sequences of cells expressing GFP-GPI not expressing AKAP9 knockout construct (top panels) and expressing AKAP9 knockout construct (magenta, bottom panels). (D) Cells transfected with AKAP9-Golgi displacement (green) and EB3-mScarlet (magenta) with cell bodies outlined with white dashed lines. Time projections show no evidence of microtubule nucleation compared with non-transfected controls (rightmost panel). All graphs displayed as mean ± SEM. ∗∗∗∗*p* < 0.0001; unpaired *t* test used for statistical analysis. Scale bars 20 μm.

To determine if Golgi associated AKAP9 is required for recruitment of γ-tubulin and axon regeneration, cells were transfected with a construct expressing an AKAP9 fragment that competitively displaces AKAP9 from the Golgi (AKAP9-dis).^18^ At 24 h post-injury *in vitro*, AKAP9-dis displaced endogenous AKAP9 away from the Golgi compared to non-transfected cells (Figure 5A; 7 transfected cells, 7 non-transfected controls; 2 animals). This was accompanied by a striking displacement of γ-tubulin away from the Golgi compared to non-transfected cells, resulting in a scattered cellular distribution of γ-tubulin puncta (Figure 5B; 9 transfected cells, 4 non-transfected cells; 2 animals). These observations were confirmed by PLA, which demonstrated loss of interactions between GM130 and AKAP9 (Figure 5A’; 56 cells; 2 animals) or γ-tubulin (Figure 5B’; 27 cells; 2 animals) in cells expressing AKAP9-dis compared to non-transfected cells (88 cells AKAP9/GM130 and 85 cells γ-tubulin/GM130; 2 animals) indicating that Golgi associated AKAP9 is required for recruitment of γ-tubulin. We next performed timelapse imaging of cells expressing AKAP9-dis and EB3-mScarlet-I to investigate the effect of displacing AKAP9 from the Golgi. This revealed a stark cessation of microtubule nucleation in cells where AKAP9 was displaced from the Golgi (Figure 5D; 6 cells; 1 animal), in contrast to cells expressing EB3-mScarlet-I only, which displayed robust microtubule nucleation at 36 h (Figure 5D; 9 cells; 4 animals) indicating that recruitment of AKAP9 and γ-tubulin to the Golgi facilitates Golgi-mediated microtubule nucleation in regenerating neurons. We then investigated if failure to recruit AKAP9 and therefore γ-tubulin to the compacted Golgi leads to errors in initiation of axon regeneration. As expression of AKAP9-dis reduced long-term viability of cells, possibly due to progressive accumulation of the AKAP9 fragment at the Golgi, and therefore precluded long-term live imaging, we performed CRISPR-mediated knockouts of AKAP9. Cells were transfected with an AKAP9 targeting CRISPR knock-out construct expressing cytoplasmic blue fluorescent protein (BFP) (px458-AKAP9-BFP), fixed at 48 h post-injury and labeled for AKAP9 and GM130 (Figure S3A). This revealed a clear reduction of AKAP9 expression in knockout cells compared to non-transfected cells (9 transfected cells, 24 non-transfected controls; 1 animal). PLA demonstrated that AKAP9 knockout resulted in loss of interactions between GM130 and γ-tubulin (Figures S3B and S3Cs; 21 cells; 3 animals) compared to non-transfected controls (25 cells; 3 animals), further indicating that AKAP9 is required for recruitment of γ-tubulin to the Golgi. We then subjected cells transfected with px458-AKAP9-BFP and GFP-GPI to live imaging starting at 24–48 h post injury. While the majority of cells not transfected with px458-AKAP9-BFP initiated axon regeneration (34/40 cells, 5 animals), cells lacking AKAP9 were unable to initiate axon regeneration up to 64 h post injury (34/36 cells, 5 animals) (Figure 5C, Videos S7 and S8; Figure S4, Videos S9 and S10). Taken together, these results demonstrate that recruitment of AKAP9 to the compacted Golgi is a critical prerequisite for recruitment of γ-tubulin, which then triggers Golgi-mediated microtubule nucleation to promote initiation of axon regeneration.

## Discussion

We demonstrate that PNI triggers dramatic fragmentation of the somatic Golgi. This is followed by rapid compaction of the Golgi and recruitment of the scaffolding protein AKAP9, which then recruits the key microtubule nucleating factor γ-tubulin during a discrete period. This regulated sequence of events ultimately results in microtubule nucleation from the compacted somatic Golgi which corresponds with initiation of axon regeneration. Importantly, this mechanism is conserved between human and rat species, highlighting the clinical relevance of these findings in PNI. Consistent with previous reports indicating that peripheral neuron regeneration proceeds at a slower pace in larger animals,^31^^,^^32^ we report corresponding differences in the timing of Golgi dynamics in rat and human neurons *in vitro*, with a 24 h delay in Golgi fragmentation in the human context. Furthermore, compared to cells *in vitro*, Golgi fragmentation is delayed by 24 h in the context of *in vivo* sciatic nerve transections, which are performed distal to the DRG cell bodies (4 cm). This delay is consistent with proximal injuries inducing a more rapid neuronal response and the reported rate of retrograde axon transport,^33^^,^^34^^,^^35^ suggesting that our observed Golgi response is initiated by the second wave of injury signaling via retrograde transport of injury-responsive transcription factors rather than the rapid retrograde calcium wave which determines neuron survival.^36^ Future work to characterize this retrograde signaling could present tangible means for the delivery of novel therapeutics to augment regeneration.

We further report that AKAP9 recruitment to the somatic Golgi is a critical prerequisite for recruitment of γ-tubulin, leading to initiation of peripheral neuron regeneration. This supports evidence that AKAP9 enhances recruitment of γ-tubulin to the Golgi.^24^^,^^25^^,^^26^ Notably, in this context γ-tubulin recruitment to the Golgi is transient *in vitro* and *in vivo*, and coincides with initiation of axon regeneration, following which γ-tubulin is again excluded from the somatic Golgi. This finding raises the possibility that, in the acute state of injury, the somatic Golgi mediated response initiates regeneration of an axon, but once this process is underway, alternative MTOC(s) coordinate maintenance of axon regeneration, perhaps through further acentrosomal nucleation sites within the regenerating axon itself. As the augmin/HAUS complex facilitates polarized microtubule nucleation in hippocampal neurons by recruiting the γ-tubulin ring complex to existing microtubules, it presents as an attractive candidate that may facilitate maintenance of polarized microtubule nucleation to sustain axon regeneration.^10^^,^^11^ Furthermore, the recently reported role of the HAUS complex in stabilizing Drosophila dendrites raises the possibility that the HAUS complex may drive an increase in microtubule density in initiating neurons to further stabilize axon extension.^12^^,^^13^ These findings therefore determine a critical temporal window during which injured peripheral neurons are competent to initiate regeneration. Novel pharmacological interventions delivered through retrograde signaling alongside timely surgical repair could potentiate more neurons at earlier time points to switch to a compacted Golgi and microtubule nucleating phenotype. Priming the innate neuronal regenerative response in this way would maximize the potential for clinically meaningful recovery of nerve function.

Interestingly, Golgi fragmentation is a hallmark of several neurodegenerative disorders such Alzheimer’s disease and Parkinson’s disease which presents before the manifestation of clinical symptoms.^37^^,^^38^ Intriguingly, neuronal repair mechanisms are impeded in these disorders, and Golgi fragmentation in this context is followed by cell death. Our work suggests that Golgi fragmentation may be a broadly conserved feature of the neuronal stress response and introduces the possibility that subsequent compaction of the fragmented Golgi may be a key first step toward initiation of repair mechanisms. In agreement with this concept, we demonstrate that induced disruption of an already compacted Golgi results in reduced AKAP9 and γ-tubulin recruitment, compromised Golgi-mediated microtubule nucleation and failed initiation of axon regeneration, further indicating that maintenance of Golgi architecture is required to sustain the initial steps of regeneration. Consequently, in addition to enhancing peripheral neuron regeneration, understanding how Golgi architecture is reestablished following neuronal stress may be critical for developing strategies for recovery of neuronal dysfunction following neurodegenerative damage. Although the mechanisms promoting Golgi compaction in the context of peripheral neuron regeneration remain unclear, a key difference in comparison to diseased neurons of the central nervous system is the proximity of satellite glial cells that promote survival of injured peripheral neurons through expression of neurotropic factors, including NGF, BDNF, and NT-3.^39^ Consistent with this, we have observed clusters of non-neuronal likely glial cells associated with regenerating peripheral neurons at the microtubule penannular stage becoming the site of axon initiation *in vitro* (Figure S1). This suggests that signaling from these cells may promote survival of injured peripheral neurons by facilitating Golgi compaction and determining axon initiation.

Overall, this work identifies sequential recruitment of AKAP9 and γ-tubulin to the compacted Golgi as a key initiator of axon regeneration in injured peripheral neurons. Furthermore, this work identifies Golgi-mediated microtubule nucleation as a conserved mechanism and potential target for therapeutic interventions aiming to potentiate the scale and rate of peripheral neuron regeneration and may inform our understanding of the mechanisms that accelerate progression of neurodevelopmental disorders.

### Limitations of the study

As this study has not assessed the functional impact of Golgi-mediated microtubule nucleation on axon regeneration *in vivo*, further studies will be required to confirm the link between the mechanisms reported here and sustained nerve regeneration in a physiologically relevant context. These *in vivo* studies are technically challenging and likely to include tissue-specific genetic manipulation to inhibit Golgi-mediated microtubule nucleation and retrograde delivery of chemical inhibitors from the site of nerve injury to prevent Golgi compaction. Furthermore, given that primary human DRG have significantly greater ethical and financial constraints than rodent cells, we were only able to obtain dissociated and fixed cells, thereby prohibiting genetic or pharmacological manipulation experiments in this context. Future functional experiments in a clinically relevant model will therefore be required to confirm the therapeutic potential of these findings.

## Resource availability

### Lead contact

Further information and requests for resources and reagents should be directed to and will be fulfilled by the lead contact, Raman M Das (raman.das@manchester.ac.uk).

### Materials availability

All reagents generated in this study are available from the lead contact without restriction.

### Data and code availability

• All data reported in this paper are available from the lead contact upon request.
• This paper does not report original code.
• Any additional information required to reanalyze the data reported in this paper is available from the lead contact upon request.

## Acknowledgments

We thank V. Allan, S. Herbert, V. Higgs, M. Houslay, M. Lowe, K. Dorey, K. Storey, and J. Wong for comments on the manuscript, P. March and S. Marsden from the University of Manchester Bioimaging Facility for technical support with microscopy and A. Adamson from the University of Manchester Genome Editing Unit for technical support with CRISPR. Funding: This work was funded by an 10.13039/501100000265MRC Clinical Research Training Fellowship MR/T028785/1, a BAPRAS Pump Priming Award 2271 PC and a University of Manchester Research Institute (UMRI) Pump Priming Award to A.E.M., R.M.D., and A.J.R. and an MRC Transition Support Fellowship MR/V036386/1 to R.M.D.

## Author contributions

Conceptualization and supervision: R.M.D. and A.J.R.; methodology: A.E.M., R.M.D., and A.J.R.; investigation: A.E.M.; visualization: A.E.M., R.M.D., and A.J.R.; writing – original draft, review and editing: A.E.M., R.M.D., and A.J.R.

## Declaration of interests

The authors declare no competing interests.

## STAR★Methods

### Key resources table

**Table:** 

REAGENT or RESOURCE	SOURCE	IDENTIFIER
**Antibodies**		

β-III-tubulin	Abcam	RRID:AB_444319
β-III-tubulin	Biolegend	RRID:AB_2315514
GM130	BD Biosciences	RRID:AB_398141
γ-tubulin	Abcam	RRID:AB_2904198
AKAP9	Sigma-Aldrich	RRID:AB_1844688
AlexaFluor®488	Abcam	RRID:AB_2732856
AlexaFluor®488	Abcam	RRID:AB_2636877
AlexaFluor®568	Abcam	RRID:AB_2636996
AlexaFluor®568	Abcam	RRID:AB_2783823
AlexaFluor®647	Abcam	RRID:AB_2890037
AlexaFluor®647	Abcam	RRID:AB_2752244
Pan-neuronal	Millipore	RRID:AB_10952564

**Biological samples**		

Primary rat dorsal root ganglia neurons	The University of Manchester (rats from Charles River Laboratories) – see below	RRID:MGI:5651135 And RRID:RGD_737932
Human dorsal root ganglia neurons	AnaBios	https://anabios.com/human-tissue-cells/human-dorsal-root-ganglia-tissue/

**Chemicals, peptides, and recombinant proteins**		

Brefeldin A	Sigma-Aldrich	B7651
Gatastatin G2	Funakoshi	FDV-0040

**Critical commercial assays**		

DuoLink proximity ligation assays	Sigma-Aldrich	https://www.sigmaaldrich.com/GB/en/product/sigma/duo92101

**Experimental models: Organisms/strains**		

Sprague-Dawley rats	Charles River Laboratories	RRID:MGI:5651135
Lewis rats	Charles River Laboratories	RRID:RGD_737932

**Recombinant DNA**		

pCAGGS-GFP-GPI	Addgene	RRID:Addgene_32601
Galt7-NeonGreen	Allele Biotech	https://reagents.allelebiotech.com/mneongreen-1/
pCAG-mScarlet-Giantin	Addgene (18)	RRID:Addgene_196872
EB3-mScarlet-I	Addgene (43)	RRID:Addgene_98826
pRD04b-AKAP9g1	This paper	Custom made in collaboration with genome editing facility at the University of Manchester
pRD04b-AKAP9g2	This paper	Custom made in collaboration with genome editing facility at the University of Manchester
pRD04b-AKAP9g3	This paper	Custom made in collaboration with genome editing facility at the University of Manchester
pBactin-AcGFP-128-425-Akap9	Addgene (18)	RRID:Addgene_196871

**Software and algorithms**		

ImageJ	ImageJ	RRID:SCR_003070
Zen Blue	Carl Zeiss	RRID:SCR_013672
Imaris	Oxford Instruments	RRID:SCR_007370
Prism	GraphPad	RRID:SCR_002798
Huygens software	Scientific Volume Imaging	RRID:SCR_014237

### Experimental model and study participant details

#### Ethics statement

Adult rats have been utilized in this study, having undertaken appropriate Home Office training. All experimental procedures adhere to the principles of replacement, reduction and refinement for use of animals in biological research and in accordance with the United Kingdom Animals (Scientific Procedures) Act 1986 under project license PP9645155.

#### Primary rat cell cultures

##### Rat dorsal root ganglia dissection and dissociation

Adult male Sprague-Dawley rats (between 8 and 12 weeks of age) were sacrificed by carbon dioxide asphyxiation followed by cervical dislocation. Spinal columns were dissected away from the animal through a dorsal longitudinal incision and subsequent steps undertaken in a laminar flow hood. Muscle and connective tissue were dissected away from the vertebral column before midline longitudinal cuts were made down the length of the vertebral column in a cranio-caudal direction on both the ventral and dorsal surfaces, such that the spinal cord and vertebral foramen were exposed. The spinal cord was gently dissected away using micro forceps under microscopic guidance. DRG were then extracted from each vertebral foramen by firm but controlled traction. Extracted DRGs were placed immediately in ice-cold F12 medium supplemented with 1% penicillin/streptomycin in a 70 mm Petri dish. Each animal yielded approximately 40 DRG. Following extraction DRG nerve roots were micro-surgically divided to leave only the body of the DRG remaining. Connective tissue and excess blood were also removed. DRGs were then dissociated to single cells for our *in vitro* model.^40^

All dissociation steps were carried out in a biological safety cabinet (class II). Explanted and cleaned DRGs were placed in a 30 mm Petri dish containing 1.8 mL warm F12 medium. 200 μL 1.25% wt/v type IV collagenase (Gibco) was then added and DRG incubated at 37°C for 60 min. The medium was removed and replaced with fresh F12 medium containing collagenase. DRG were incubated for a further 45 min at 37°C. Collagenase solution was removed and DRG washed three times with 2 mL warm F12. 1.8 mL F12 was then added along with 200 μL 2.5% wt/v trypsin (Gibco) and DRG incubated for 30 min at 37°C. Trypsin was then removed and 1.5 mL F12 with 500 μL foetal bovine serum (FBS) was added to stop trypsinisation. DRG were again washed three times with 2 mL F12 and after the final wash a further 2 mL F12 was added and DRG transferred using a Pasteur pipette to a 15 mL falcon tube. DRG were triturated by gently pipetting up and down using a Pasteur pipette 8 to 10 times. Remaining undissociated ganglia/debris were allowed to settle and supernatant containing dissociated cell suspension transferred to a fresh 15 mL falcon. A further 2 mL F12 was added to undissociated DRG and the process of trituration and supernatant transfer repeated until all DRG were dissociated. The resultant dissociated cell suspension was passed through a sterile 70 μm mesh to remove debris then centrifuged at 900 rpm for 5 min at 37°C. To achieve a neuron-rich culture a 15% bovine serum albumin (BSA) solution was prepared by combining 500 μL BSA with 500 μL F12. This solution was pipetted down the side of a clean 15 mL falcon such that a ‘track’ was formed over which the cell solution would be applied. After centrifugation, the supernatant was removed, and cells resuspended in 500 μL F12. The cell suspension was pipetted into the BSA containing falcon along the previously formed ‘track’ and centrifuged at 1200 rpm for 10 min at 37°C. Cells were then either resuspended in nucleofector solution, in the case of transfection (see below), or in an appropriate volume of F12 supplemented with N2 for immunofluorescence staining. Cells were plated onto either 13 mm 1.5 glass coverslips in 4-well plates or FluoroDish 35 mm dishes ready coated with poly-D-lysine (PDL) (World Precision Instruments). Glass coverslips were coated with 0.1 μg/μl PDL for 20 min and washed once with F12. Glass coverslips and/or FluoroDishes were then coated with laminin, prepared 1:100 in F12 (to reach a final concentration of 1–2 μg/cm^2^). Plates/dishes were incubated for 2 to 24 h at 37°C. After coating, the laminin solution was removed, and glass washed once with F12 prior to cell plating.

#### *In vivo* animal studies

##### *In vivo* rat sciatic nerve injury

Adult male Lewis rats between 8 and 12 weeks of age were used for *in vivo* study due to the lower incidence of autotomy following nerve transection. All animals were acclimatised for at least 1 week prior to surgery and housed in groups of at least 2 with standard feeding and care as per the University of Manchester protocols. Littermates were randomly assigned to experimental groups. Animals were anesthetized using isofluorane inhalation anesthesia. Induction was carried out in an induction chamber with 2 L/min oxygen flow and 5% isofluorane until animals lost their righting reflex and breathing slowed. Maintenance of anesthesia was achieved using a nose cone and 2–2.5% isofluorane. Throughout surgery depth of anesthesia was monitored regularly by assessing breathing rate/depth and pedal withdrawal reflex. 20 min prior to the initiation of surgery, all animals received subcutaneous buprenorphine analgesia (0.3 mg/mL stock) at a dose of 0.01 mg/kg and the surgical area was minimally shaved. Surgery was carried out using an aseptic technique and animals placed on a 37°C heat pad to minimize heat/fluid loss. The sciatic nerve was exposed through a dorsal gluteal approach and transection performed at the level of the proximal femur. Animals were allowed to recover for: 0 h, 24 h, 48 h and 7 days before undergoing terminal anesthesia by overdose of isofluorane followed by cervical dislocation. L4 and L5 DRG were then extracted and immediately fixed in 4% PFA for 4 h at room temperature.

#### Primary human cell cultures

##### Primary human dorsal root ganglia

Human DRG were obtained from AnaBios (USA). Cadaveric DRG from consenting donors with no comorbidities were extracted, dissociated, cultured and fixed by the suppliers according to their optimized protocols before being shipped to our lab. Cytosine arabinose at a concentration of 10 μM was requested to be added to the cell culture medium to reduce the density of non-neuronal cells. Cells were cultured on glass bottom 96-well plates on which immunofluorescence labeling was performed.

### Method details

#### Dissociated rat DRG electroporation

Dissociated DRG were resuspended in 100 μL Lonza nucleofector solution (brought to room temperature 30 min before use). For optimal transfection 1 million cells were required, which equates to the DRG from one whole rat. A total of 1–2 μg (1 μg per construct) DNA plasmid solution was then added to the cell suspension and gently mixed by pipetting up and down. The suspension was then transferred to a sterile Lonza nucleofection cuvette, ensuring that the suspension contained no air bubbles and was at the bottom of the cuvette. Program G-013 was selected on the Nucleofector device and the cuvette inserted into the carrier before performing electroporation. Following electroporation, 500 μL of warmed supplemented media was added to the cuvette and transferred carefully using the supplied Lonza plastic Pasteur pipette to a sterile Eppendorf tube. The resultant suspension was then incubated at 37°C for 10 min to aid cell recovery from electroporation. During the recovery time warmed supplemented media was applied to prepared coverslips/FluoroDishes (to result in 150 μL per 13 mm coverslip and 600 μL per FluoroDish) and these dishes incubated at 37°C until plating. Without repeated aspirations, the cell suspension was then divided between the prepared plates/FluoroDishes (4 times the volume of suspension was added to FluoroDishes when compared to 13 mm coverslips). The cells were incubated at 37°C for 2 h, after which media containing residual nucleofector solution was removed and fresh supplemented media added (F12 with 1:100 N2; 600 μL per well in four well plates and 2 mL per FluoroDish). Media was subsequently changed three times per week.

#### Plasmids

The following plasmids were used: pCAGGS-GFP-GPI, Galt7-NeonGreen,^41^ pCAG-mScarlet-Giantin (a gift from Frank Bradke, Addgene plasmid # 196872^18^), EB3-mScarlet-I (a gift from Dorus Gadella, Addgene plasmid # 98826^42^), pRD04b-AKAP9g1, pRD04b-AKAP9g2, pRD04b-AKAP9g3 (AKAP9 CRISPR knock out constructs designed and produced in collaboration with The University of Manchester genome editing core facility), pBactin-AcGFP-128-425-Akap9 (a gift from Frank Bradke, Addgene plasmid # 196871^18^) at a concentration between 500 ng and 1 μg per construct, not exceeding 2 μg per transfection.

#### Fixation and immunofluorescence labeling of dissociated DRG cells

Dissociated DRGs were fixed at the following time points: 2 h, 16 h, 24 h, 48 h and 7 days (rodent cells) and 24 h, 48 h and 72 h (human cells) post-injury. For each time-point, experimental triplicates were used. Cells were fixed with 4% paraformaldehyde (PFA) for 20 min at room temperature. Following fixation, cells were washed once with phosphate buffered saline (PBS) for 5 min. Prior to staining, PBS was removed and cells permeabilised using 0.2% Triton X-100 for 30 min at room temperature, followed by a PBS wash. Blocking was then carried out using normal donkey serum (Abcam) diluted 5:100 in antibody diluent (prepared by adding 1 g BSA, 1 g sodium azide and 300 μL Triton X-100 to 1 L PBS). Following blocking cells were then incubated with primary antibodies (concentrations listed in table below) for either 2 h at room temperature or 16 h at 4°C. Cells were then washed in PBS once for 5 min. PBS was removed and secondary antibodies (table below) diluted 1:500 in antibody diluent were applied and plates incubated at room temperature for 1 h in the dark. Following incubation, cells were washed with PBS once and counterstained for DAPI (1:1000) for 3 min followed by a final PBS wash. Coverslips were mounted onto glass Superfrost slides (Thermo Fisher Scientific) using either ProLong Diamond or ProLong Glass antifade mounting medium (Thermo Fisher Scientific) slides/dishes were stored at 4°C both prior to, and following, imaging.

#### Fixation and immunofluorescence labeling of DRG explants

L4 and L5 DRG from *in vivo* experiments were extracted and immediately fixed in 4% PFA for 4 h at room temperature. DRG were washed with PBS and then dehydrated in 30% sucrose solution for 24 h (or until DRG sunk). They were then embedded in OCT and snap frozen on dry-ice before being placed at −80°C prior to sectioning. Sectioning was carried out on a Leica CM3050s cryostat and 20 μm sections obtained. Immunofluorescence staining was carried out as for *in vitro* cells.

**Table:** 

Primary antibodies					
Target	Clonality	Host species	Immunogen	Supplier (reference)	Concentration
β−ΙΙΙ−tubulin	Polyclonal	Rabbit	Synthetic peptide corresponding to Human b-III-tubulin aa 350 to the C-terminus	Abcam (Ab18207)	1:1000
β−ΙΙΙ−tubulin	Monoclonal	Mouse	Microtubules derived from rat brain	Biolegend (MMS-435P)	1:1000
GM130	Monoclonal	Mouse	Rat GM130 aa. 869-982	BD transduction (610822)	1:250
γ−tubulin	Monoclonal	Rabbit	Recombinant fragment	Abcam (Ab179503)	1:200
AKAP9	Polyclonal	Rabbit	Recombinant Protein Epitope Signature Tag (PrEST) antigen sequence	Atlas antibodies (HPA026109)	1:200

Secondary antibodies				
Fluorophore	Host species	Target species	Supplier (reference)	Concentration
AlexaFluor®488	Donkey	Mouse	Abcam (ab150105)	1:500
AlexaFluor®488	Donkey	Rabbit	Abcam (ab150073)	1:500
AlexaFluor®568	Donkey	Mouse	Abcam (ab175472)	1:500
AlexaFluor®568	Donkey	Rabbit	Abcam (ab175470)	1:500
AlexaFluor®647	Donkey	Mouse	Abcam (ab150107)	1:500
AlexaFluor®647	Donkey	Rabbit	Abcam (ab150075)	1:500

Conjugated antibodies					
Target	Fluorophore	Host species	Immunogen	Supplier (reference)	Concentration
Pan-neuronal	AlexaFluor®488	Rabbit	Whole Neuron Marker	Sigma-Aldrich (ABN2300A4)	1:200

Antibodies used in this study.

#### Proximity ligation assays

Duolink Proximity Ligation Assay (PLA) kits were used according to manufacturer’s instructions. Fixed DRG on 13 mm glass coverslips were used at the following time-points: 16 h, 24 h, 48 h and 7 days. Samples were first permeabilised using 0.2% Triton X-100 for 30-min at room temperature. Blocking was then carried out using 40 μL Duolink blocking solution per coverslip. Coverslips were incubated in a humidity chamber at 37°C for 60 min. Blocking solution was then removed and replaced with 40 μL primary antibodies (GM130 and AKAP9 or GM130 and γ-tubulin) diluted in Duolink antibody diluent. Samples were incubated in a humidity chamber for 2 h at room temperature or 16 h at 4°C. Following primary antibody incubation, the solution was removed, and coverslips washed twice with 1x Duolink wash buffer A at room temperature (5 min per wash). 40 μL PLA PLUS and MINUS probes diluted 1:5 in Duolink antibody diluent were then added to each coverslip and samples incubated in a humidity chamber at 37°C for 60 min. Probes were then removed, and coverslips washed twice with wash buffer A. 1x Duolink ligation buffer was prepared by diluting the 5x buffer 1:5 with high purity water, then ligase added to the resultant solution at 1:40 immediately prior to addition to coverslips. Wash buffer was removed and 40 μL ligation solution was applied. Samples were incubated in a humidity chamber at 37°C for 30 min. Ligation solution was removed, and samples washed twice with wash buffer A. Amplification buffer was then prepared by diluting 5x amplification buffer 1:5 in high purity water and polymerase added immediately prior to use at 1:80 in amplification buffer. 40 μL of resultant solution was added to each coverslip and samples incubated for 100 min at 37°C. Amplification solution was removed, and samples washed twice with 1x wash buffer B at room temperature (10 min per wash). A final 1 min wash was carried out using 0.01x wash buffer B. To prepare the samples for imaging, the remaining wash buffer was removed, and coverslips mounted onto glass slides using Duolink *in situ* mounting medium (containing DAPI) and edges sealed with clear nail varnish.

#### Pharmacological treatments

*In vitro* DRG were treated at 23 h post-injury and either underwent timelapse imaging or fixation after 1 h of treatment for immunofluorescence staining with either 30 μM Gatastatin G2 (Funakoshi), 5 μg/mL BFA (Sigma-Aldrich), or control DMSO 1% diluted in F12 supplemented with 1:100 N2. In the case of EB3 imaging, cells were treated at 36 h post-injury.

#### Fixed cell and tissue imaging

Images were acquired using a Zeiss Cell Observer Z1 microscope system (Carl Zeiss) equipped with a Colibri7 light-emitting diode (LED) illumination system and 63 ×1.4 numerical aperture (NA) objective and Flash4 v2 sCMOS camera (Hamamatsu). ZenPro 2.3 blue edition (Carl Zeiss) software was used for image acquisition and post-acquisition processing. Images were deconvolved using a constrained iterative algorithm, maximum 40 iterations and 0.1% quality threshold. Higher resolution was achieved for select cells using STED microscopy using a Leica Microsystems TCS SP8 STED system equipped with a 100 ×1.4 NA oil immersion STED objective. Images were acquired using a 488 nm excitation laser and 592 nm depletion laser for the green channel and a 568 nm excitation laser and 660 nm depletion laser for the red channel. Z sections were separated by 0.2 μm and images were scanned at 10 Hz using 2x line averaging. The resulting images were deconvolved using Huygens Professional (Scientific Volume Imaging). For *in vivo* transection experiments, matched tissue sections from L4/L5 DRG were taken from age/strain matched rats and underwent identical fixation, sectioning and staining protocols for immunofluorescence and PLA.

#### Time-lapse imaging

Time-lapse imaging was performed using a widefield Zeiss Axio-observer microscope system equipped with a Colibri7 LED illumination system (Carl Zeiss), Flash4 v2 sCMOS camera (Hamamatsu) and heated chamber with 5% carbon dioxide. For assessment of cell dynamics (GFP-GPI transfected cells), images were acquired every 10 to 20 min for 10 to 24 h using a 40×1.2 NA silicone immersion objective. For EB3 (EB3-mScarlet transfected cells) images were acquired as fast as possible for 1 to 3 min using a 63×1.4 NA objective. Z plane intervals were 0.5 μm and minimal exposure times (20-50 ms) were used in each channel. Time-lapse images were deconvolved in ZenPro using a fast iterative algorithm, 0.1% quality threshold and a maximum of 40 iterations.

#### Image analysis

To perform analysis of Golgi disconnected components Imaris 9.2.1 software (Bitplane) was used. The surfaces function was used with automatic thresholding to reduce the risk of bias. This enabled 3-dimensional (3D) analysis of Golgi structure, including the number of discrete components, termed ‘number of disconnected components’.

Imaris was also used to quantify PLA puncta using the spot function. This enabled each distinct fluorescent signal to be picked up as a single spot. The number of spots and the volume of puncta occupying each cell were then quantified. The spot manual tracking function was also used to visualise EB3 comet trajectory.

### Quantification and statistical analysis

#### Statistical analysis

GraphPad Prism 9 (GraphPad Software, California, USA) was used to carry out statistical analysis. To quantify data for Golgi apparatus disconnected components, as well as PLA spot analysis, a one-way analysis of variance (ANOVA) with Tukey’s multiple comparison test was used. *p* < 0.05 was considered statistically significant. Unpaired T tests were used when comparing two groups with one independent variable and *p* < 0.05 was considered statistically significant in each case. Statistical details of experiments can be found in results section main text and figure legends. ∗*p* < 0.05, ∗∗*p* < 0.01, ∗∗∗*p* < 0.001, ∗∗∗∗*p* < 0.0001 and graphs displayed as mean ± SEM.

## Supplemental information

Document S1. Figures S1–S4

Video S1. Timelapse sequence of regenerating neuron expressing GFP-GPI (greyscale) at 24–40 h post-injury

Video S2. Timelapse sequence of regenerating neuron expressing Galt7-NeonGreen (red) and EB3-mScarlet-I (green) imaged at 36 h post injury for 2 min

Video S3. Timelapse sequence of regenerating neuron expressing Galt7-NeonGreen (red) and EB3-mScarlet-I (greyscale) imaged at 36 h post injury before and following application of BFA

Video S4. Timelapse sequence of regenerating neuron expressing GFP-GPI (green) imaged in medium containing BFA at 24–40 h post-injury

Video S5. Timelapse sequence of regenerating neuron expressing GFP-GPI (green) imaged in medium containing Gatastatin G2 at 24–40 h post-injury

Video S6. Timelapse sequence of regenerating neuron expressing GFP-GPI (green) imaged in medium containing DMSO at 24–40 h post-injury

Video S7. Timelapse sequence of regenerating neuron expressing GFP-GPI (green) but not transfected with AKAP9 knockout construct at 24–40 h post-injury

Video S8. Timelapse sequence of regenerating neuron expressing GFP-GPI (green) and AKAP9 knockout construct (magenta) at 24–40 h post-injury

Video S9. Timelapse sequence of regenerating neuron expressing GFP-GPI (green) but not transfected with AKAP9 knockout construct at 48–64 h post-injury

Video S10. Timelapse sequence of regenerating neuron expressing GFP-GPI (green) and AKAP9 knockout construct (magenta) at 48–64 h post-injury
