# Supplementary material for: Golgi-mediated microtubule nucleation is associated with initiation of vertebrate peripheral neuron regeneration
Source: iScience. 2025 Oct 4;28(11):113697. doi: 10.1016/j.isci.2025.113697 (PMC12590029; doi:10.1016/j.isci.2025.113697)
Supplement: Document S1. Figures S1–S4 [file mmc1.pdf]

iScience, Volume *28*

Supplemental information

Golgi-mediated microtubule nucleation is associated with initiation of vertebrate peripheral neuron regeneration

Alice E. Mortimer, Adam J. Reid, and Raman M. Das

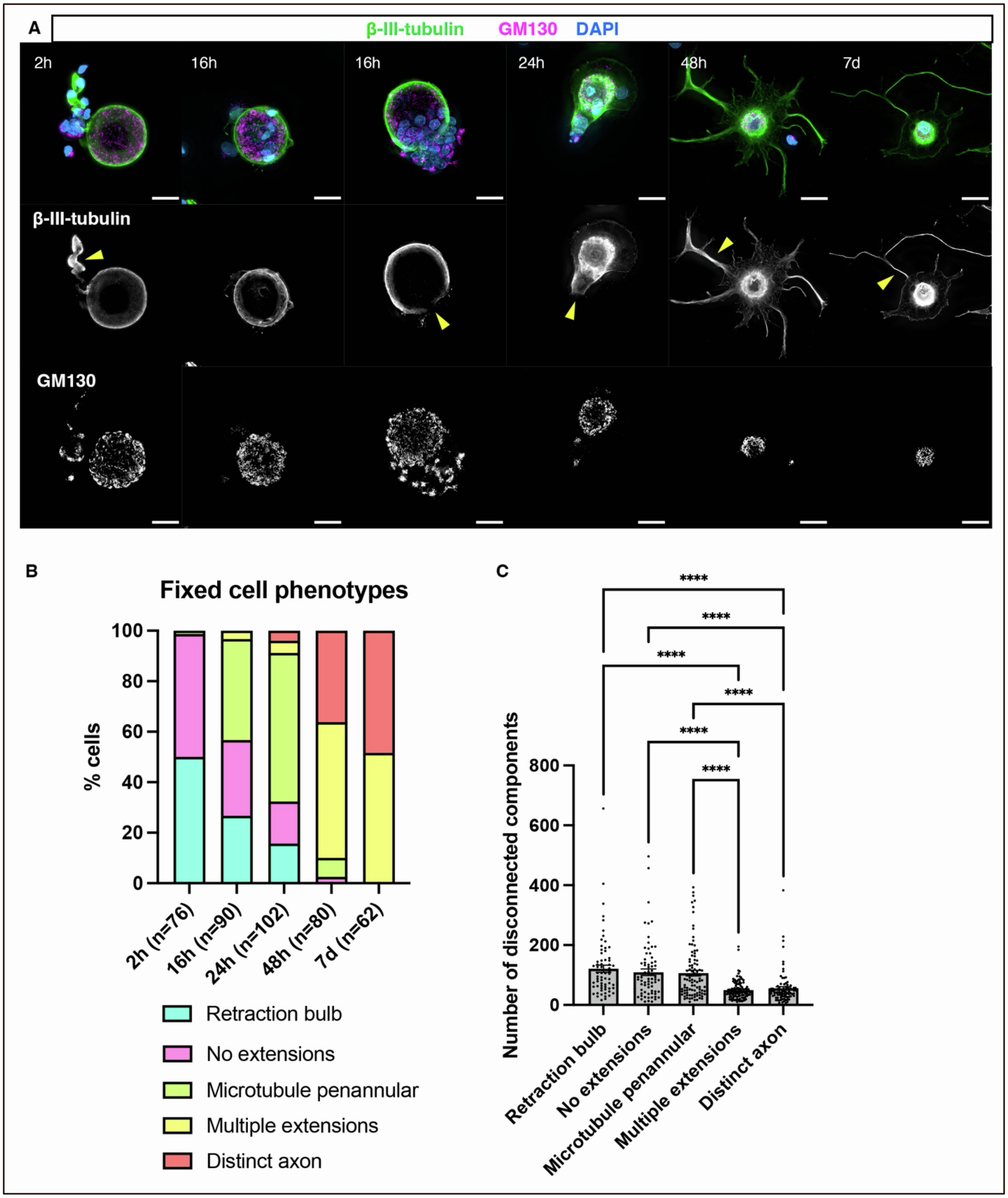

Fig. S1. Golgi Compaction is associated with initiation of axon regeneration.

A) Immunostaining to detect neuron specific microtubules (β-III-tubulin) and GM130 from 2 hours up to 7 days post-injury. Yellow arrowheads: retraction bulb at 2 hours; the microtubule penannular at 16 hours; initiation of regeneration at 24 hours; multiple extensions at 48 hours; and a distinct axon at 7 days. **B)** Quantification of the percentage

of cells at observed stages of regeneration at each time point. **C)** Quantification of mean number of disconnected components in GM130 channel according to cell phenotype: 120.8 ± 11.98 with retraction bulbs (n=78 cells), 109.1 ± 1.08 with no extensions (n=83 cells), 106.5 ± 9.26 with microtubule penannular (n=103 cells), 49.36 ± 2.94 with multiple extensions (n=83 cells) and 55.34 ± 5.71 with a distinct axon (n=63 cells). N=3 animals throughout. p=<0.0001 retraction bulb vs multiple extensions; p=<0.0001 retraction bulb vs distinct axon; p=<0.0001 no extensions vs multiple extensions; p=<0.0001 no extensions vs distinct axon; p=<0.0001 microtubule penannular vs multiple extensions; p=<0.0001 microtubule penannular vs distinct axon.**** $p \leq 0.0001$, ordinary one-way ANOVA and Tukey's post hoc test used for statistical analyses. Scale bars: 20 μm.

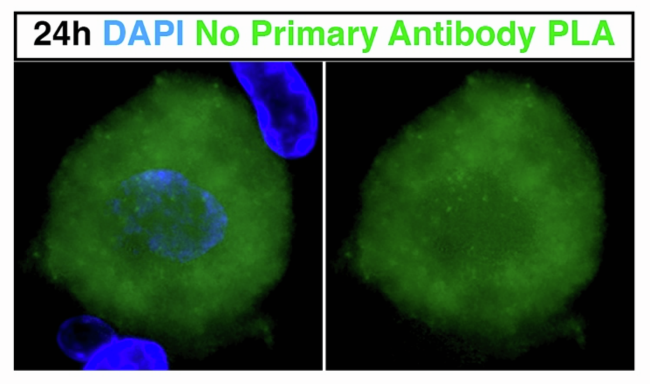

Fig. S2. Negative control for proximity ligation assays. PLA carried out on cells at 24 hours post-injury in the absence of primary antibodies for GM130, AKAP9 or γ-tubulin.

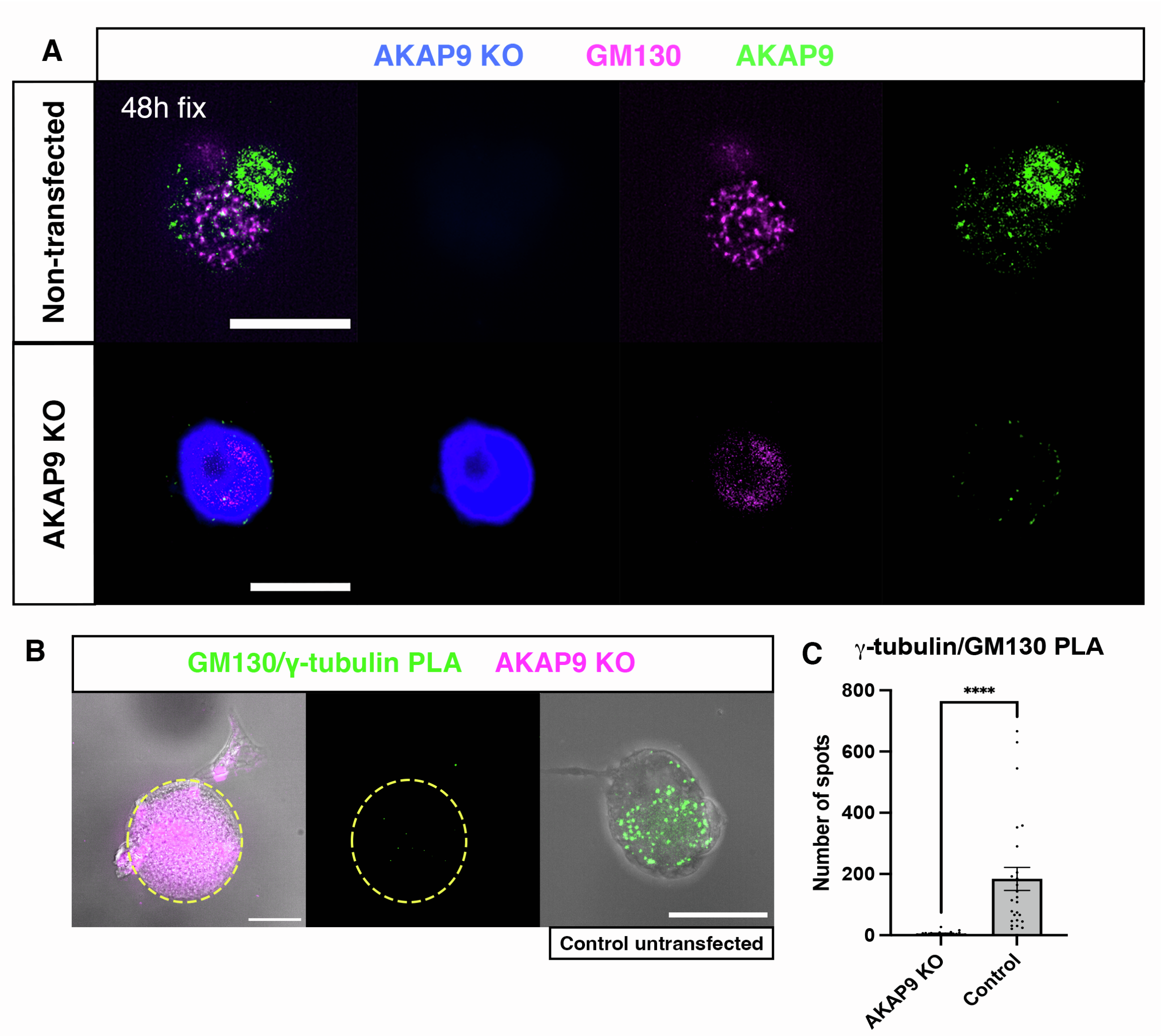

Fig. S3. AKAP9 knock out reduces AKAP9 expression and Golgi γ-tubulin associations. A) Control non-transfected cells (top panels) and AKAP9 KO cells (bottom panels) fixed at 48 hours post injury. **B)** AKAP9 KO cells (magenta) fixed at 24 hours post injury, followed by PLA to determine interactions between GM130 and γ-tubulin (green). Yellow dashes outline the cell in the leftmost panel. The rightmost panel shows a control non-transfected cell. **C)** Quantification of PLA puncta: 5.67 ± 1.36 in AKAP9 knock out cells (N=3 animals, n=21 cells), 184.2 ± 37.75 in control non-transfected cells (N=3 animals, n=25 cells). ****$p<0.0001$, unpaired T test used for statistical analysis. Scale bars: 20 μm.

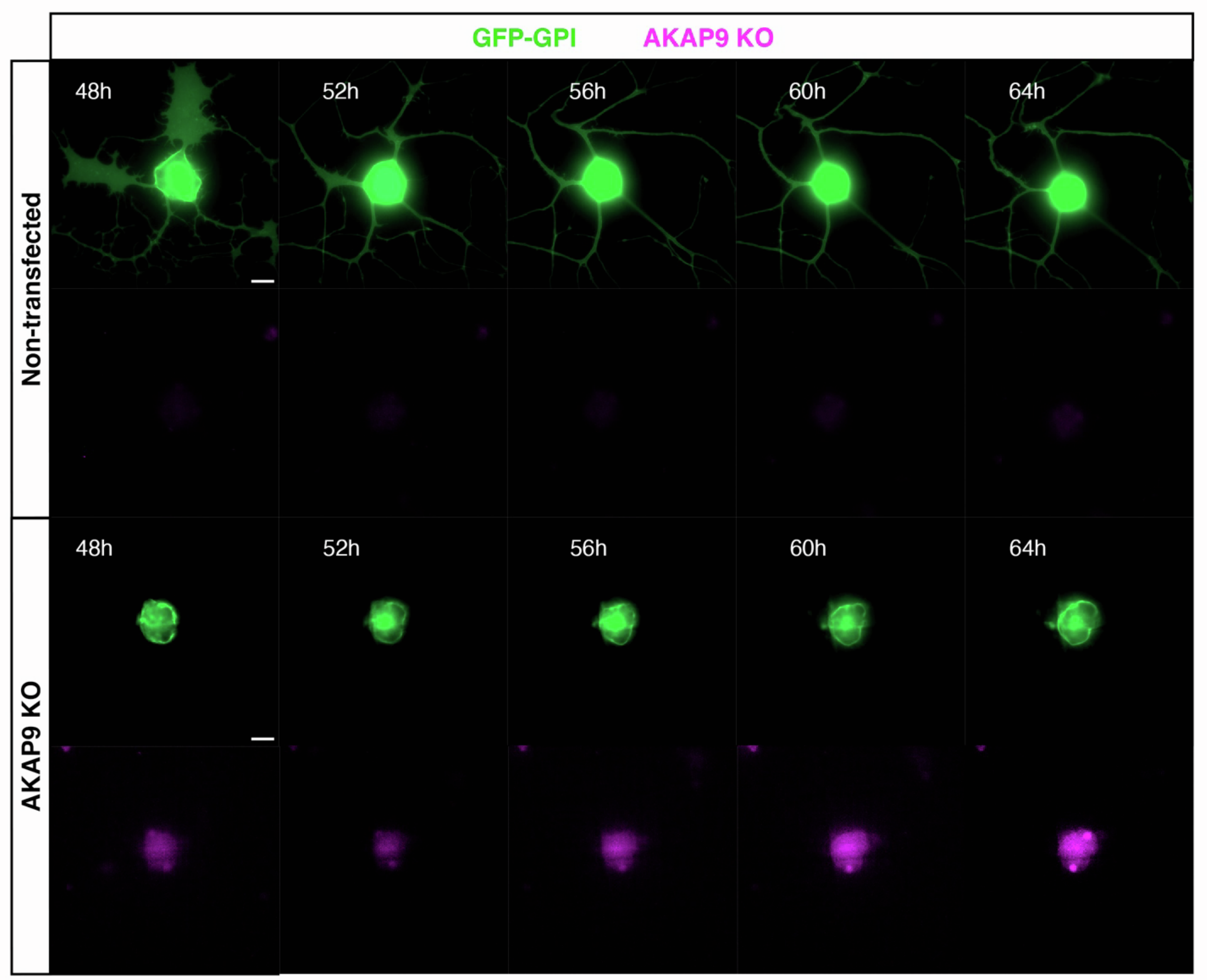

Fig. S4. AKAP9 Knock out results in compromised axon regeneration from 48 to 64 hours following injury. Timelapse images of cells expressing GFP-GPI to label the cell membrane (green) from 48 to 64 hours. Top panel shows a control cell extending an axon and bottom panel a cell expressing AKAP9 knock out construct (magenta). Scale bars: 20 μm.
